# Supplementary material for: The Journey to Sustainable Participation in Physical Activity for Adolescents Living with Cerebral Palsy
Source: Children (Basel). 2023 Sep 10;10(9):1533. doi: 10.3390/children10091533 (PMC10528208; doi:10.3390/children10091533)
Supplement: Supplementary file 1 [file children-10-01533-s001.zip › children-2587497-supplementary.pdf]

Table S1 Interview Schedule

Participant	Interview 1			Interview 2			Interview 3		
	Setting	Attended by	Interviewed independently	Setting	Attended by	Interviewed independently	Setting	Attended by	Interviewed independently
A1, M<br>13 y	Home	Mother	No	Training location	Mother	No	Home	Mother	No
A2, M<br>13 y 1 mo	School	Mother	No	Home	Father	Yes	Home	Father	Yes
A3, M,<br>13 y, 11 mo	Zoom	Mother and father	No	Testing location	Father	No	Home	Father	No
A4, F<br>14 y, 6 mo	Home	Mother	Yes	Testing location	Mother	Yes	Testing location	Mother	Yes
A5, M<br>15 y 6 mo	School	Mother	No	Home	Mother	No	Home	Mother	No
A6, M<br>11 y 2 mo	Home	Mother and father	No	Training location	Mother	No	Home	Mother and father	No
A7, M<br>14 y 4 mo	Home	Mother	Yes	Testing location	Mother	Yes	Testing location	Mother	Yes
A8, M<br>15 y 8 mo	Cafe	Mother and father	No	Testing location	Father	No	Not available		

Note. Training location was at the high school grounds where the high-level mobility programme was conducted. The testing location was at an indoor child development centre. *Abbreviations.* A, adolescent; M, male; F, female; y, age in years; mo, months.
